# In vitro antiviral activity of twenty-seven medicinal plant extracts from Southwest Nigeria against three serotypes of echoviruses

**DOI:** 10.1186/s12985-018-1022-7

**Published:** 2018-07-18

**Authors:** Omonike O. Ogbole, Toluwanimi E. Akinleye, Peter A. Segun, Temitope C. Faleye, Adekunle J. Adeniji

**Affiliations:** 1 Department of Pharmacognosy, Faculty of Pharmacy, University of Ibadan, Ibadan, Nigeria; 2 Department of Pharmacognosy, Faculty of Pharmacy, Olabisi Onabanjo University, Sagamu, Nigeria; 3 Department of Microbiology, Ekiti State University, Ado-Ekiti, Nigeria; 4 Department of Virology, College of Medicine, University of Ibadan, Ibadan, Nigeria

**Keywords:** Antiviral activity, Cytopathic effect inhibition, Echoviruses, *Macaranga barteri*, Nigerian plants

## Abstract

**Background:**

Echoviruses, a serotype of enteroviruses, infect millions of people globally and there is no specific drug treatment or vaccine available for its management. The screening of medicinal plants used locally for the treatment of infectious diseases, can provide a reliable option in the discovery of potent therapeutic compounds. This study was carried out to investigate the antiviral activities of 27 medicinal plant extracts, belonging to 26 different plant species, selected from Nigerian ethnobotany, against echovirus 7, 13 and 19 serotypes (E7, E13 and E19, respectively).

**Methods:**

The plants were macerated in methanol and the cytotoxicities of the crude extracts were evaluated on the rhabdomyosarcoma cell line using the MTT assay. The antiviral activity of the plant extracts and fractions against echoviruses (E7, E13, and E19) was determined using the neutralisation assay, an assay that measures the inhibition of cytopathic effect on cell culture.

**Results:**

The crude extract of *Macaranga barteri* leaves had the highest cytotoxicity with CC_50_ value of 0.27 μg/mL. This was followed by *Crinum jagus* (9.88 μg/mL) and *Terminalia ivorensis* (12.14 μg/mL). The antiviral screening showed that ten out of the 27 crude plant extracts tested were active on E7 and E19, inhibiting the cytopathic effect of the virus in tissue culture. None of the extracts inhibited the cytopathic effect caused by E13 serotype. Amongst the active plant extracts, the methanol extract of *M. barteri* leaves had the highest antiviral activity on both E7 and E9 with IC_50_ values of 0.028 and 0.0017 ng/mL, respectively, followed by the *Ageratum conyzoides* extract (0.208 μg/mL, E7; 0.006 μg/mL, E19) and *Mondia whitei* extract (0.038 μg/mL, E7; 0.005 μg/mL, E19). Amongst the fractions of *M. barteri, the* DCM fraction was most the active and selective on E7 (IC_50_ = 0.0075 ng/mL; SI = 19,896.54) and E19 (IC_50_ = 0.0175 ng/mL; SI = 8581.24).

**Conclusion:**

Our research has demonstrated that *Macaranga barteri* extracts has potent antiviral activity against echoviruses E7 and E19, and our findings suggest that this extract may have potential as a therapeutic agent in the treatment of enteroviral infections.

## Background

The use of traditional medicine is popular in Africa, with almost three-quarter of the populace of this continent consulting traditional medical practitioners (TMPs), mainly traditional doctors, when faced with a medical problem. This is mainly because traditional healthcare system is easily accessible, culturally acceptable and comparatively cheaper to the costly orthodox medicine. In Nigeria and most developing countries, medicinal plants are traditionally used to treat a variety of ailments, especially infectious diseases [1].

Although medicinal plants have been widely regarded as a constant source of safe and effective medicines with potential to yield newer drugs, this potential is under threat due to the alarming biodiversity loss, with recent estimates indicating that every fifth plant species on earth is threatened with extinction [2]. Hence, scientists in drug discovery are making urgent effort to document and research into bioactivity of medicinal plants used in various ethnobotany, in order to establish correlation between the ethnomedical usage of medicinal plants and drug discovery in modern medicines [3, 4].

Enteroviruses (EVs) are single positive-stranded genomic RNA viruses of the family Picornaviridae that consists of more than 100 serotypes. They have emerged as one of the important etiological agents for encephalitis, especially in children and adults. For instance, enteroviruses such as coxsackievirus A9, A10 and B5; echoviruses 4, 5, 9, 11, 19 and 30; and EV 71, 75, 76 and 89, from various parts of the world have been reported in encephalitis cases and epidemics [5]. Echoviruses (enteric cytopathic human orphan viruses) are small (~ 300 Å in diameter - 27 nm), non-enveloped icosahedral viruses which belong to enterovirus species B of the family Picornaviridae with 29 serotypes (E1–E29). The most frequent clinical diseases of echoviruses include fever of short duration and sometimes a rash or mild upper respiratory symptoms [6]. Clinical syndromes associated with infections by echoviruses include aseptic meningitis, paralysis, encephalitis, ataxia, Guillain-Barré syndrome, exanthema, respiratory disease, diarrhoea, pericarditis, myocarditis and hepatic disturbance. Echoviral infection, like other enteroviruses, occurs via faecal-oral route transmission [7].

There is no specific drug treatment in clinical use available for enteroviruses, and at the moment, development of vaccine against all EVs is not feasible due to the great number of serotypes [8, 9]. Added to this is the general problem of viral resistance towards antiviral agents, especially RNA viruses, which can mutate at high frequencies and evolve rapidly into drug-resistant strains [10]. Therefore, the increasing need for search of new effective compounds with anti-enteroviral activity is imperative. Natural products from medicinal plants have proved to be effective against a wide variety of viral diseases by inhibiting the replication cycle of various DNA and RNA viruses [11]. This validated the use of plant-based antivirals as an integral part of many traditional systems of medicine [12]. Therefore, this work aimed at determining the antiviral activities of 27 plant extracts against echovirus 7, 13 and 19 serotypes.

## Methods

### Plant materials and extraction

Twenty-seven different morphological parts from 26 plants (Table 1), selected based on their ethnobotanical use in the treatment of infectious diseases, were collected from various locations in Ibadan, South-west Nigeria, identified and authenticated at Forestry Herbarium Ibadan (FHI). Plant parts used were air-dried, pulverized and extracted by maceration in methanol for 72 h at room temperature (26–33 °C). The resulting extracts were filtered and concentrated in vacuo using the rotary evaporator.

**Table 1:** Plant species analysed for antiviral activity

S/N	Family	Name	Local Name (Yoruba)	Part Used	Voucher (FHI) No	MNTC (μg/mL)	Ethnomedicinal uses
1	Amaryllidaceae	*Crinum jagus* (J. Thomps.) Dandy	Isu-meri	bu	109011	10	Tuberculosis, epilepsy, asthma, infections, anti-snake venom and sickle cell diseases [24].
2	Anacardiaceae	*Spondias mombin* L.	Iyeye	l	108861	10	Stomach ache, abdominal discomfort, diabetes, wound healing, haemorrhoids and vermifuge [25].
3	Anacardiaceae	*Spondias mombin* L.	Iyeye	ba	108861	10	Stomach ache, abdominal discomfort, diabetes, wound healing, haemorrhoids and vermifuge [25].
4	Apocynaceae	*Picralima nitida* (Stapf) T. Durand & H. Durand	Abeere	s	108794	1	Hypertension, fever, jaundice, dysmenorrhea, malaria and abdominal discomfort [26].
5	Asclepiadaceae	*Secamone afzelii* (Roem et Schult) K. Schum	Ailu	l	109995	1	Diabetes, colic, dysentery, hypertension and malaria [27].
6	Asteraceae	*Ageratum conyzoides* L.	Imi-esu	l	106040	100	Purgative, ulcers, mental illness, infections, skin diseases, wound healing, febrifuge, pneumonia, toothache and rheumatism [28].
7	Boraginaceae	*Heliotropium indicum* L.	Apari-igun	l	110156	10	Wounds, flatulence, inflammation, skin ulcers and conjunctivitis [29].
8	Combretaceae	*Terminalia ivorensis* A. Chev.	Afara-dudu	ba	105432	1	Syphilis, burns, bruises, arthritis and haemorrhoids [30].
9	Combretaceae	*Terminalia superba* Engl. & Diels.	Afara-funfun	ba	109800	1	Diabetes, skin infections, leg ulcers and cancers [31].
10	Convolvulaceae	*Ipomoea asarifolia* (Desr.) Roem. & Schult.	Gboro-ayaba	l	110052	1	Skin infections, abdominal cramps and diarrhoea [32].
11	Crassulaceae	*Bryophyllum pinnatum* (Lam.) Oken	Abamoda	l	109487	100	Worm expellant, cold, pneumonia and respiratory tract infections [33].
12	Cucurbitaceae	*Lagenaria breviflora* (Benth.) Roberty	Tagiri	s	109040	1	Female infertility, measles, chicken pox and skin infections [34].
13	Dilieniaceae	*Tetracera alnifolia* Willd.	Opon	l	107511	10	Constipation, cancer, leprosy, toothache and cough [35].
14	Euphorbiaceae	*Croton gratissimus* Burch.	Ajekobale	l	109041	1	Coughs, bleeding gums, eye disorders, influenza, fever, malaria, skin infections [36].
15	Euphorbiaceae	*Macaranga barteri* Mull. Arg.	Agbaasa	l	107230	0.01	Gonorrhoea, syphilis, skin infections, cancer and burns [37].
16	Lamiaceae	*Hoslundia opposita* Vahl	Efirin-odan	l	100866	10	Burns, skin infections, cough and malaria [38].
17	Leguminosae	*Calliandra portoricensis* (Jacq.) Benth	Tude	r	109672	0.1	Prostate cancer, inflammations, cough and haemorrhoids [39].
18	Meliaceae	*Entandrophragma utile* (Dawe & Sprague) Sprague	Jebo	ba	86848	1	leg ulcer, anthelmintic, wound, abdominal pain [40].
19	Mimosaceae	*Mimosa pudica* L.	Patanmo	l	100332	0.1	Fevers, piles, jaundice, leprosy and dysentery [41].
20	Nyctaginaceae	*Boerhavia diffusa* L.	Etipase-erinla	r	109603	1	Diabetes, cancer, inflammation, infections and epilepsy [42].
21	Periplocaceae	*Mondia whitei* (Hook.f.) Skeels	Isirigun	l	110043	100	Malaria, diabetes, infertility and erectile dysfunction [43].
22	Periplocaceae	*Parquentina nigrescens* (Afzel) Bullock	Ogbo	l	108852	1	Abdominal cramps, gonorrhoea, rickets and asthma [44].
23	Phytolaccaceae	*Petiveria alliacea* L.	Awogba	l	100512	100	Pain, influenza, cold, diabetes, malaria and skin infections [45].
24	Piperaceae	*Piper guineensis* Schumach	Iyere	l	110051	10	Sickle cell anaemia, male infertility, inflammation and skin infections [46].
25	Poaceae	*Eleusine indica* (L.) Gaertn.	Gbegi	l	92140	1	Diabetes, anthelminthic, cough and wound [47].
26	Rubiaceae	*Sarcocephalus latifolius* (Sm.) E. A. Bruce	Egbesi	l	109707	10	Epilepsy, diarrhoea, dysentery, malaria and fever [48].
27	Verbenaceae	*Lippia multiflora* Moldenke	Eforomoba	l	108858	10	Fever, constipation, ear infections, eye troubles, and diabetes [49].

*ba* bark, *bu.* bulb, *l* leaves, *s* seed, *r* root

### Fractionation of the active extract

The most active crude extract (*Macaranga barteri*) was subjected to bioassay guided fractionation using liquid-liquid partitioning and vacuum liquid chromatography (VLC). Briefly, 150 g of the crude methanol extract of *M. barteri* was dissolved in 250 mL of 70% aqueous methanol and the mixture was partitioned successively in *n*-hexane (400 mL × 4), dichloromethane (DCM; 400 mL × 4), ethylacetate (EtOAc; 400 mL × 4) and aqueous methanol (400 mL × 4). As the DCM fraction displayed the highest antiviral activity, it was subjected to vacuum liquid chromatography (VLC) on silica gel using a gradient elution solvent system that comprised of *n-*hexane/EtOAc (10:1 to 5:5, each 250 mL). Based on the similarity of their analytical TLC profiles, the fractions obtained were pooled into six sub-fractions (DCMA – DCMF). The VLC fractions were sprayed with chromogenic spray reagents including 1% vanillin/sulphuric acid and aluminium chloride (AlCl_3_) to detect the classes of possible compounds present.

### Viruses and cell line

Three serotypes of echovirus (E7, E13, and E19) used in this study were obtained from stool isolate at the WHO Polio Laboratory, Department of Virology, University of Ibadan, Nigeria. All viruses were stored at − 70 °C until use. The human rhabdomyosarcoma (RD) cells were obtained by Centre for Disease Control, Atlanta, Georgia. The cells were grown in Eagle’s minimum essential medium (MEM; Sigma-Aldrich) supplemented with 10% fetal bovine serum (FBS), 100 units/mL of penicillin, 100 μg/mL of streptomycin, 2 mM L-glutamine, 0.07% NaHCO_3_, 1% non-essential amino acids and vitamin solution. The cells were cultured in 37 °C in 5% CO_2_ humidified incubator for 72 h. The test medium used for cytotoxic assays and antiviral assays contained only 2% of fetal bovine serum (maintenance medium).

### Preparation of stock solution of extract and fraction

Crude extracts and fractions, 10 mg each were dissolved in 1 mL dimethlysulfoxide (DMSO) and filtered through a sterile syringe filter (0.2 μm pore diameter) to obtain a stock solution (SS; 10 mg/mL). The SS was diluted with the maintenance medium (culture medium that contained only 2% FBS) to obtain a final concentration of 1000 μg/mL (0.1% DMSO).

### Tissue culture infective dose (TCID)

Virus titres were determined by virus-induced cytopathic effect in RD cell culture and were expressed as 50% tissue culture infective concentration (TCID_50_) per mL using Sperman-Karber’s method. Briefly, RD cells were seeded into T25 cell culture flask (Corning®, UK), and incubated at 37 °C/5% CO_2_ environment for 24 h. Thereafter, 100 μL of E7 were incorporated into the T25 flask containing the cultured RD cells and incubated for 72 h, leading to an increment in the virus stock quantity as a result of the 100% cytopathic effect. One hundred microliters RD cell suspension (1 × 10^6^ cells/mL) was seeded into three 96-well microtitre plates and incubated for 24 h to form monolayer. Ten-fold serial dilutions of the virus stock was made and 100 μL of each dilution was inoculated into the wells. A well that contained only RD cells without any virus served as the cell control. The microtitre plate was incubated at 37 °C, and daily CPE scoring was done till the control wells started dying. The TCID_50_ values was determined using Spearman-Karber’s method and 100 TCID_50_ was used for the assay. The procedure was done in triplicate and repeated for the E13 and E19.

### Cytotoxicity assay

The MTT (3-(4,5-dimethythiazol-2-yl)-2,5-diphenyl tetrazolium bromide) colorimetric assay, which is known to be a reliable measure of cell viability, was used to determine the cytotoxicity of the plant extracts and fractions. The principle of this assay involves the reduction of yellow MTT dye by mitochondrial succinate dehydrogenase to an insoluble, coloured (dark purple) formazan product. The purple formazan-containing cells are then solubilized with an organic solvent such as DMSO to release the solubilized formazan reagent which is measured by spectrophotometry. The assay was carried out based on the protocol described in an earlier literature [13]. In brief, ten-fold serial dilutions (1000 to 0.01 μg/mL) of the extracts / fractions were made with the maintenance. Monolayers of RD cells were seeded into each well of a 96-well microplate and treated with various concentrations of each extract/fraction. Plates were incubated at 37 °C in 5% CO_2_ humidified incubator for 72 h. Plates were then observed under the microscope for cell death/cytopathic effect (CPE). The minimum dilution of extract with no toxic effect on the cells was referred to as the maximum non-toxic concentration (MNTC). After the incubation period, the old medium was removed and 25 μL of MTT solution (2 mg/mL) was added to each well and incubated for 2 h at 37 °C. thereafter, the MTT solution was removed from the wells and DMSO (75 μL) was added to dissolve formazan crystal. Optical density was measured spectrophotometrically (Multiscan 347, MTX lab) at 490 nm. The experiment was conducted in triplicate and the 50% cytotoxic concentration (CC_50_) was calculated.

### Antiviral activity assay

The antiviral activity of the plant extracts and fractions against echoviruses (E7, E13, and E19) was determined using the neutralisation assay, an assay that measures the inhibition of cytopathic effect on cell culture. The method previously described in the literature was modified and used in this assay [14]. Briefly, RD cells were seeded in a 96-well microplate and incubated for 24 h to grow to confluency. After the incubation period, 50 μL of 100 TCID_50_ virus suspension were added to confluent cell monolayers in a 96-well plate and allowed to stand for 1 h to enable virus adsorption. Thereafter, ten-fold serial dilutions, obtained from the MNTC of each extract were added in triplicate into all the wells with the exception of the negative control wells that contained only RD cells and the virus control that contained an equal virus concentration but lacked the plant extract. The plates were incubated at 37 °C in 5% CO_2_ humidified incubator for 72 h after which the cell viability was measured using the MTT assay as described above. The concentration of the plant extract that decreased the CPE by 50%, with respect to the virus control, was estimated from the obtained optical density and defined as the 50% inhibition concentration (IC_50_). The selectivity index (SI), defined as CC_50_ over IC_50,_ for each active extract and fractions were also determined. Since there are no antiviral drugs approved for the treatment of enteroviral infections, no drug control was used in this study. All experiments were carried out in triplicates.

### Data analysis

The experiments were conducted in triplicate. The 50% cytotoxic concentration (CC_50_) and the 50% inhibitory concentration (IC_50_) for the extracts and fractions were calculated from concentration-effect-curves after linear regression analysis using GraphPad Prism5 and Microsoft Excel. The selectivity index for each active extract and fractions were also determined. Selective index is a comparison of the amount of a test agent that causes the inhibitory effect to the amount that causes toxicity.

## Results

### Tissue culture infective dose

The determination of the virus titre (TCID_50_) for E7, E13, and E19 viruses by Sperman-Karber’s method gave a value of 10^− 6^ for the three viruses. Therefore, 100 TCID_50_ which was used for the study was calculated as 10^− 4^.

### Maximum non-toxic concentration (MNTC) of crude extracts

The plant extracts encountered in this study displayed varying MNTC to RD cells in tissue culture medium. As presented in Table 1, the extract of *Macaranga barteri* leaves had the lowest MNTC (0.01 μg/mL), while *Petiveria alliacea*, *Mondia whitei*, *Ageratum conyzoides* and *Bryophyllum pinnatum* extracts had the highest MNTC of 100 μg/mL.

### Cytotoxic activities of plant extracts and fractions

As presented in Table 2, the cytotoxic concentration that inhibited 50% of cell growth (CC_50_) showed that the crude extract of *M. barteri* leaves had the highest cytotoxicity with CC_50_ value of 0.27 μg/mL. This was followed by *Crinum jagus* (9.88 μg/mL) and *Terminalia ivorensis* (12.14 μg/mL). The DCM fraction of *M. barteri* showed the highest cytotoxicity amongst the fractions with CC_50_ value of 0.18 μg/mL. Other fractions including *n*-hexane, ethyl acetate and aqueous had CC_50_ values of 24.9, 2.87 and 3.01 μg/mL, respectively. Amongst the VLC sub-fractions of *M. barteri*, sub-fraction A (DCMA) displayed the highest cytotoxicity on the RD cells with CC_50_ value of 0.47 μg/mL (Table 3).

**Table 2:** Anti-echovirus activity of crude methanol extracts

S/N	Plant	CC_50_ (μg/mL)	IC_50_ (μg/mL)			SI	
			E7	E13	E19	E7	E19
1	*Ageratum conyzoides*	155.33	2.08*10^− 3^	NA	5.64*10^− 4^	746.78	2.80*10^4^
2	*Bryophyllum pinnatum*	125.47	3.13	NA	2.03	40.07	61.96
3	*Crinum jagus*	9.88	0.21	NA	1.94	46.65	5.09
4	*Ipomoea asarifolia*	84.21	2.52*10^−3^	NA	NA	35,004	–
5	*Lippia multiflora*	112.07	1.532	NA	NA	73.15	–
6	*Macaranga barteri*	0.27	2.76*10^−5^	NA	1.69*10^−6^	7971.00	1.30*10^5^
7	*Mondia whitei*	132.50	0.04	NA	5.35*10^−4^	3447.83	2.4*10^4^
8	*Spondias mombin* ^a^	53.33	0.10	NA	NA	512.3	NA
9	*Terminalia ivorensis*	12.14	0.01	NA	NA	1213.4	NA
10	*Tetracera alnifolia*	147.8	1.61	NA	NA	91.7	NA

*NA* Not Active, *SI* Selectivity Index; ^a^ Stem back extract

**Table 3:** Anti-echovirus activity of *M. barteri* fractions and sub-fractions

S/N	Fractions	CC_50_ (μg/mL)	IC_50_ (μg/mL)			SI	
			E7	E13	E19	E7	E19
1	Hexane	24.9	0.44	NA	0.31	57.96	83.85
2	DCM	0.18	7.54*10^−6^	NA	1.75*10^−6^	19,865.54	8581.24
3	DCMA	2.97	0.09	NA	0.54	33.82	5.50
4	DCMB	0.89	0.03	NA	0.05	28.21	16.38
5	DCMC	0.63	NA	NA	0.01	–	54.59
6	DCMD	1.31	4.80*10^−3^	NA	0.01	247.70	246.1
7	DCME	0.47	0.02	NA	0.01	24.23	44.09
8	DCMF	24.35	1.61	NA	1.82	15.13	13.36
9	EtOAc	2.87	NA	NA	NA	–	NA
10	Aqueous	3.061	NA	NA	NA	–	NA

*NA* Not Active, *SI* Selectivity Index

### Antiviral screening of plant extracts and fractions

The preliminary antiviral screening showed that only ten out of the 27 crude plant extracts tested were active on E7 and E19, inhibiting the cytopathic effect of the virus in tissue culture (Table 2). On E7, *M. barteri* displayed 75% CPE inhibition at MNTC (0.01 μg/mL), 50% inhibition at 0.001 μg/mL, while the extract exhibited 75% CPE inhibition at MNTC (0.01 μg/mL) and 50% CPE inhibition at 0.01 ng/mL on E19. None of the extracts inhibited the cytopathic effect caused by E13 serotype. Amongst the active plant extracts, the crude methanol extract of *M. barteri* leaves had the highest antiviral activity on both E7 and E9 with IC_50_ values of 0.028 and 0.0017 ng/mL, respectively, followed by the *Ageratum conyzoides* extracts (0.208 μg/mL, E7; 0.006 μg/mL, E19) and *Mondia whitei* extracts (0.038 μg/mL, E7; 0.005 μg/mL, E19) (Table 2). Amongst the liquid-liquid partitioned fractions of *M. barteri*, the DCM was most the active and selective on E7 (IC_50_ = 0.0075 ng/mL; SI = 19,896.54) and E19 (IC_50_ = 0.0175 ng/mL; SI = 8581.24) when compared with the hexane fraction which had moderate activity on E7 (IC_50_ = 0.442 μg/mL; SI = 57.96) and E19 (IC_50_ = 0.305 μg/mL; SI = 83.85). However, both the ethyl acetate and aqueous fractions of *M. barteri* were inactive against E7 and E19. In addition, sub-fraction D (DCMD) displayed the highest antiviral activity amongst the sub-fractions on both E7 (IC_50_ = 0.00477 μg/mL), and E19 (IC_50_ = 0.00532 μg/mL) (Table 3).

### Detection of compounds in the active *M. barteri* sub-fraction

Yellow colouration and mint green spots were observed when sub-fraction D (DCMD) was sprayed with vanillin/sulphuric acid. In addition, yellow spots were observed at short (254 nm) and long (365 nm) wavelengths of UV light when the sub-fraction was sprayed with aluminium chloride.

## Discussion

Enteroviruses have continued to pose a great burden to global health. Its faecal-oral route of transmission has enhanced the spread of these infectious agents, especially in developing nations, where there is poor sanitation and hygiene. Since the indigenes of these low and middle-income countries utilise medicinal plants to treat diseases caused by viruses, it is therefore imperative to investigate the antiviral potentials of indigenous medicinal plants.

In this study, 27 plant extracts selected from Nigerian ethnobotany were screened for their anti-enteroviral activity and we identified ten extracts with potent inhibitory activity against E7 and E19. Generally, the cytotoxic and anti-echovirus activities of all the extracts, partitioned or VLC fractions in this work were observed to be concentration dependent. The leaf extract of *Macaranga barteri* demonstrated the highest cytotoxic activity (CC_50_ = 0.27 μg/mL), antiviral activity (IC_50_ = 0.028 ng/mL on E7; 0.00167 ng/mL on E19) and selectivity index (SI = 7971 on E7; 130,177.50 on E19). It was observed that the cytotoxicity evaluation of the extracts seemed to correspond directly to their anti-echovirus activities, a finding similar to our earlier result [15]. *Ipomoea asarifolia* leaf extract also showed potent antiviral activity against E7 (IC_50_ = 0.0025 μg/mL) with a high selectivity index (SI = 35,004) but was inactive against E19. Other plants with potent antiviral activity encountered in this study include *Mondia whitei*, *Ageratum conyzoides* and *Terminalia ivorensis*.

The most active plant extract, *M. barteri* was subjected to liquid-liquid partitioning and the DCM fraction showed the highest cytotoxicity (CC_50_ = 0.18 μg/mL) and anti-echovirus activity (IC_50_ = 0.0075 ng/mL on E7; 0.0175 ng/mL on E19) with very high selectivity index (SI = 19,896.54 on E7; 8581.24 on E19). Thus, it is safe to state that the anti-enteroviral property of *M. barteri* lies majorly in the moderately polar DCM fraction. The DCM fraction of some *Macaranga* species have been shown to be responsible for various bioactivity observed in the genus, especially against bacteria, cancer cells and *Plasmodium falciparum* [16, 17]. Fractionation of the DCM fraction using VLC led to a reduction in both the cytotoxic and antiviral activity (Table 3). This suggests the possibility of synergistic effect in the DCM partitioned fraction, prior to VLC fractionation.

*Macaranga barteri*, the most active of the 11 crude extracts, belongs to the family Euphorbiaceae, and it is used in Nigeria ethnomedicine as vermifuge and to treat dysentery (a gastroenteric infection) which could be caused by virus, bacteria or parasitic worm infections [18]. Therefore, the anti-enteroviral activity demonstrated by *M. barteri* in this study could justify the ethnomedicinal use of this plant in Nigeria. In addition, antimicrobial activity has been identified and generally implicated in the genus *Macaranga* [19]. An earlier research by Cos and co-workers reported that the leaves extract of another member of the *Macaranga* genus, *M. kilimandscharica* displayed high anti-measles activity, alongside a potent antiviral activity against Herpex simplex type 1 and Coxsackie viruses [20].

On spraying the most active sub-fraction with aluminium chloride, yellow spots were observed which indicated the presence of flavonoids. Flavonoids (especially prenylated flavonoids) and stilbenes have been reported as the major constituents of *Macaranga* and are said to be most likely responsible for most of the bioactivities of this genus [16]. Also, a wide range of flavonoids has been shown to possess antiviral activities against a variety of RNA viruses such as poliovirus, sindbis virus, respiratory syncytial virus (RSV), and DNA virus such as herpes simplex virus (HSV) [21]. The proposed antiviral mechanisms of action of flavonoids include inhibition of viral polymerase and binding of viral nucleic acid or viral capsid proteins [15]. Previous HPLC profiling of the DCM extract of *M. barteri* leaves revealed 3,5-dicaffeoylquinic acid, acteoside, kampferol-7-*O*-glucoside and bastadin-11 as its major constituents [17]. Kaempferol-7-*O*-glucoside is a flavonol glucoside which possessed potent antiviral activity against *Herpes simplex* virus (HSV) and HIV-1 [22]. 3,5-dicaffeoylquinic acid (DCQA) is a phenolic acid which has been reported as a good inhibitor of HIV integrase, an enzyme required for integration of viral DNA into cellular DNA. Its antioxidant and anti-inflammatory activities have also been reported [23]. The anti-infective activities (antiviral and antimicrobial) of 3,5-dicaffeoylquinic acid (DCQA), acteoside, kampferol-7-*O*-glucoside which had been reported in literature and are present in DCM partitioned fraction of *M. barteri* could support the anti-echovirus activity of *M. barteri* reported in this study.

The relatively high selectivity of the *M. barteri* extract and fractions, especially the most active sub-fraction (SI = 274.70 on E7; 246.10 on E19) could be attributed to minimal toxicity possessed by the supposedly constituted flavonoids since flavonoids are believed to possess minimal toxicity, being distributed in edible plants and beverages [15].

## Conclusion

To summarize our findings, we have reported the antiviral activity of 26 medicinal plants selected from Nigerian flora against three serotypes of enteroviruses (E7, E13 and E19). Ten of the medicinal plant extracts exhibited potent antiviral activity against E7 and E19, with *Macaranga barteri* being the most active. In addition, the DCM fraction *M. barteri* displayed the most potent antiviral activity amongst the fractions. Further research is in progress to isolate and elucidate the bioactive components that may be responsible for the antiviral activity of this plant and to determine their mechanism of action. To the best of our knowledge, this is the first report of the antiviral activity of *M. barteri* and indicates that the ethnobotanical use of these drugs may provide some benefits in the treatment of infectious diseases, thereby warranting further investigation.

## Abbreviations

CC_50_: 50% cytotoxic concentration
CPE: Cytopathic effect
DCM: Dichloromethane
DMSO: Dimethlysulfoxide
E13: Echovirus 13
E19: Echovirus 19
E7: Echovirus 7
EtOAc: Ethyl acetate
FBS: Fetal bovine serum
FHI: Forestry herbarium Ibadan
IC_50_: 50% inhibition concentration
MNTC: Maximum non-toxic concentration
MTT: 3-(4,5-dimethythiazol-2-yl)-2,5-diphenyl tetrazolium bromide
RD: Human rhabdomyosarcoma cells
SI: Selectivity index
TCID_50_: 50% tissue culture infective concentration
TMPs: Traditional medical practitioners
VLC: Vacuum liquid chromatography
